# Spanish Nursing Students’ Attitudes toward People Living with HIV/AIDS: A Cross-Sectional Survey

**DOI:** 10.3390/ijerph17228672

**Published:** 2020-11-22

**Authors:** María Adelaida Álvarez-Serrano, Encarnación Martínez-García, Adelina Martín-Salvador, María Gázquez-López, María Dolores Pozo-Cano, Rafael A. Caparrós-González, María Ángeles Pérez-Morente

**Affiliations:** 1 Department of Nursing, Faculty of Health Sciences, University of Granada, 51001 Ceuta, Spain; adealvarez@ugr.es; 2 Department of Nursing, Faculty of Health Sciences, University of Granada, 18016 Granada, Spain; emartinez@ugr.es (E.M.-G.); pozocano@ugr.es (M.D.P.-C.); rcg477@ugr.es (R.A.C.-G.); 3 Guadix High Resolution Hospital, 18500 Guadix, Granada, Spain; 4 Department of Nursing, Faculty of Health Sciences, University of Granada, 52005 Melilla, Spain; 5 Mind, Brain and Behavior Research Center (CIMCYC), Faculty of Psychology, University of Granada, 18016 Granada, Spain; 6 Department of Nursing, Faculty of Health Sciences, University of Jaén, 23071 Jaén, Spain; mmorente@ujaen.es

**Keywords:** attitudes, HIV/AIDS, students, nursing

## Abstract

Human immunodeficiency virus (HIV) infection is still a public health issue. Human immunodeficiency virus/acquired immune deficiency syndrome (HIV/AIDS) creates, in society, stigmatizing attitudes, fear, and discrimination against infected people; even health professionals do not feel trained enough to adequately take care of these patients, which affects the quality of care provided to such patients. The purpose of this study was to explore nursing students’ attitudes and other related factors toward people with HIV/AIDS, as well as their evolution in subsequent academic years. A cross-sectional study was performed with students in four academic years from four Spanish health sciences institutions (*n* = 384). Data were collected voluntarily and on an anonymous basis, utilizing the “Nursing students’ attitudes toward AIDS” (EASE) validated scale. The students’ attitudes toward people with HIV/AIDS were relatively positive, with a total mean EASE value of 85.25 ± 9.80. Statistically significant differences were observed according to the academic year (*p* = 0.041), in 4 out of 21 items of the scale and among students with no religious beliefs. By adjusting every variable, only the weak association with religion was maintained (*p* = 0.045).

## 1. Introduction

Human immunodeficiency virus (HIV) infection is still an international public health problem [1,2]. In 2018, an estimated 37.9 million people worldwide were infected, of which 24.5 million people were treated with antiretroviral medications by mid-2019 [3]. In this sense, the World Health Organization, within the Sustainable Development Goals for the year 2030, included as a global goal to reduce HIV infection to zero contagions, zero discrimination, and zero deaths caused by the virus [2,4].

Today, particularly in developed countries, people with HIV live longer and experience the disease as a chronic condition, the most common comorbidities being cardiovascular diseases, kidney diseases, psychiatric disorders, and neoplasias, mainly associated with aging [3,5,6]. Currently, having HIV/AIDS still has a significant impact on the economic, political, legal, and social levels [1,2]. Regarding the latter, it has been documented that HIV/AIDS generates, among people, stigmatizing attitudes, fear, and discrimination against infected persons [7].

Furthermore, with the passing of time, as the disease settles in society, knowledge and education about HIV/AIDS have been reduced [8]. In the field of health, several authors state that even healthcare workers who are more exposed to work accidents with biological materials [9], such as nursing students and professionals, do not feel prepared enough to treat such patients adequately [6,8,10,11]. Variables such as ideology, age, stress, and negative opinions on homosexuality affect healthcare professionals’ attitudes toward people with HIV/AIDS [1]. In addition, the lack of knowledge and the existence of erroneous concepts as regards transmission of HIV not only foster stigmatizing attitudes and fear of contagion, but they also impact clinical practices related to HIV/AIDS and the quality of the care received by seropositive patients [6,10,12,13,14]. In relation to these attitudes, in a study carried out by Valencia et al., it was reflected that women with HIV felt that they were mistreated by health professionals and that their rights to confidentiality and privacy were violated [15], which undoubtedly undermines legislation and ethics of care [12,13]

With respect to the assessments of HIV/AIDS patients performed by nursing students, there is research that affirms that these negative evaluations are related to stigmas related to promiscuity, drug addiction, and homosexuality [8,16,17]. In order to change such perceptions, many research contributions have shown that an increase in knowledge in this area reduces fear and anxiety [6,11].

Although it is reasonable to think that nursing students’ negative attitudes toward people with HIV/AIDS will diminish as their academic level increases, there is a discrepancy in scientific literature regarding such a hypothesis [1,18,19]. Therefore, the objective of this study is to explore nursing students’ attitudes and other related factors toward HIV/AIDS and their evolution in subsequent academic years.

## 2. Materials and Methods

### 2.1. Data Collection

An online, cross-sectional survey was conducted anonymously by self-administration. The participants were nursing students from four Spanish health sciences, institutions with a four-year-long study program of 240 credits. Although these four institutions are located in different geographical areas, student´s sociocultural levels of similar. The collection of the questionnaires was administered simultaneously in the four institutions during one month of the 2019–2020 academic year. It was sent through the teaching support platform, and participation was voluntary outside of class hours through the teaching support platform. The members of the research group invited all students enrolled across the four academic years to participate. After the first dissemination of the questionnaire, a reminder was sent to those students who had not yet answered the survey.

### 2.2. Measurement Tool

For data collection, the “Nursing students’ attitudes toward AIDS” (EASE) one-dimensional scale was employed, the Spanish version of which was initially created and validated by Tomás-Sábado (Appendix A) [20]. This tool showed good internal validity in the identification of nursing students’ attitudes toward HIV/AIDS in Spain (Cronbach’s alpha coefficient = 0.779). This scale comprises 21 items with Likert-type responses ranging from 1 = totally agree to 5 = totally disagree. The score for each item depends on its directionality. Items 3, 5, 7, 8, 11, 14, 15, and 21 are scored from 5 to 1, 5 being totally agree and 1 being totally disagree, and items 1, 2, 4, 6, 9, 10, 12, 13, and 16–20 are scored from 1 to 5, 1 being totally agree and 5 being totally disagree. The scale’s maximum score is 105, which indicates the most positive attitudes, and the minimum score is 21, which indicates the most negative and preconceived attitudes. For each participant, the scale’s global value was calculated by adding each item’s score (with a total of 21 scores). The original scale was used in most of the studies analyzed [1,21,22]. Additionally, the following sociodemographic variables were considered: age, sex (male or female), academic year, marital status (in a relationship or not in a relationship), sexual orientation (heterosexual or non-heterosexual), and religion (with religious beliefs or with no religious beliefs).

### 2.3. Data Analysis

Prior to data analysis, the internal consistency of the scale was assessed by determining Cronbach’s alpha, with a satisfactory result of 0.776. Next, a descriptive analysis of the entire sample was carried out by means of absolute and relative frequencies and the calculation of measures of central tendency (i.e., median and standard deviation) according to the quantitative or qualitative nature of the study’s variables. The scale’s normality of values was explored, and as a result of not being possible to verify their parametricity, for the inferential statistics, a nonparametric analysis was adopted. Differences among the scale’s values according to the sociodemographic variables and academic year categories were analyzed through the Mann–Whitney *U* test, and for variables with more than two factors, the Kruskal–Wallis *H* test was employed. In cases of significant differences among academic years, post-hoc multiple comparisons were carried out through Bonferroni correction. The effect size was calculated using Kerby´s formula [23]. To establish a correlation between age and the final score of the EASE scale, Spearman’s Rho was used. Finally, the influence of the sociodemographic variables and academic year on the EASE scale’s final score was explored by using a multiple linear regression model through the “introduce” method, in which the academic year, re-categorized into three dummy variables, was included, taking the first year as reference. Data were treated with the Statistical Package for the Social Sciences (SPSS) program, version 25, (IBM, New York, NY, USA, for Windows).

### 2.4. Ethical Considerations

This study complies with the good clinical practice regulations, as stated in the European Directive 2001/20/CE and Law 14/2007, of 3 July, on biomedical research. Treatment of personal data in health research is governed by Organic Law 3/2018, of 5 December, on Data Protection and Guarantee of Digital Rights. Every participant checked a box indicating consent to participate in the study.

## 3. Results

The sample included 384 students, with the response rate being 18%. Twenty-nine percent of the sample (*n* = 115) were in their first year of study. With regards to sex, 84% were women, and the total mean age was 23 ± 6.7 years. In terms of academic year, the mean age was significantly higher for those in their fourth year of study (*p* < 0.001). With respect to students’ sexual orientation, 337 (87.8%) were heterosexual, a higher proportion of which was observed in the third year of study (*p* = 0.012). Regarding relationship status, 207 (53.9%) stated they were not in a relationship, the fourth year of study being the year with a higher number of students in a relationship (*p* = 0.008). Of the total number of students, 265 (69%) considered themselves as having religious beliefs (Table 1).

The total EASE score in the analyzed sample was 85.25 ± 9.80. Table 2 shows such values according to sociodemographic variables and academic year. Among the second-year students and among those with no religious beliefs, the highest statistically significant scores were observed (*p* = 0.041, and *p* = 0.006, with an effect size of 0.17, respectively) (Table 2). Significant causes according to academic year were identified in the second and first year of study (*p* = 0.050 with an effect size of −0.22) (results not shown).

The scale items that reached a higher mean score were P1 (“AIDS does not affect heterosexual couples”) with 4.62 ± 1.01 and P11 (“being an AIDS carrier should not be a barrier to accessing education and employment”) with 4.68 ± 0.87. Such items show the highest percentages of “disagree” and “agree” answers, with 83.1% and 83.9%, respectively (results not shown).

With respect to the questions asked in the scale, there were significant differences among academic years in 4 out of the 21 items included, namely in item 2 (“fetuses infected with AIDS should be aborted”), 3 (“there is no risk arising from AIDS carriers’ use of restaurants and pubs”), 4 (“seropositive women should not be allowed to get pregnant”), and 16 (“in hospitals, AIDS carriers should not share a room with non-infected persons”) (Table 3). The causes for the significance among academic years in item 2 were observed between the first and second years (*p* = 0.041), between the first and third years (*p* = 0.0001), and between the first and fourth years (*p* = 0.021); in item 3, between the first and second years (*p* = 0.022); in item 4, between the first and third years (*p* = 0.018); and in item 16, between the first and second years (*p* = 0.014) (results not shown). In all cases, the first-year score was lower than that obtained in the subsequent years.

In the multiple linear regression model, it was observed that by adjusting all variables, only religion was associated with the scale values, with those stating no religious beliefs showing more favorable attitudes toward HIV/AIDS ((95% CI: 0.05–4.26); *p* = 0.045) (Table 4).

## 4. Discussion

The attitudes toward people with HIV/AIDS of the students that participated in the study were relatively positive or favorable, since they indicated a mean EASE score above 85 points, as recommended by Tomás-Sábado and Aradilla-Herrero [1]. This result coincides with that reported in several similarly designed studies [1,21,24], reinforcing the idea that nursing, as a profession, through its humanistic approach, generates among future professionals positive attitudes toward an infection such as HIV or a disease such as AIDS [25].

According to the academic level, some differences were found in the EASE scores, the first academic year’s score being lower than those obtained in subsequent years. Such a result coincides with that published by Leyva-Moral et al., where the lowest percentage of positive attitudes was observed among first-year students [25]. Nevertheless, differences in scores were found between the second- and first-years, but not in subsequent years with respect to the first one, which contradicts previous studies regarding an increase in favorable attitudes as the academic level increases [1,18].

Some authors have stated that when facing problems that are especially sensitive at a social level and that are ideologically charged, such as HIV and AIDS, an increase in knowledge alone is not enough to change people’s beliefs or attitudes [22,24,25,26,27]. Furthermore, contact with actual patients occurring in subsequent years during clinical practice, on the contrary, could increase fear of contagion among students, thus obstructing an advance toward higher levels of disease tolerance, which is an issue that requires further investigation [22].

The median age of the analyzed sample was in accordance with those published in similar studies [1,9,21,22,24,25,28], corresponding to young adults, although this variable was not associated with attitudes toward HIV/AIDS. However, the observed tendency, that as age increased, attitudes became more negative, coincides with the results of other studies that confirmed such an association [25,29,30,31].

Despite the fact that the analysis was reconducted to assess the EASE scale’s internal consistency by obtaining a Cronbach’s alpha satisfactory result, some items showed a change opposite to the one expected. That is, it was expected that in cases of positive assertions the level of agreement would increase and in cases of negative assertions, the level of disagreement would increase. However, in terms of item 14, “persons infected with AIDS should be considered victims of the social system,” 66.4% of students disagreed or totally disagreed, and for item 10, “seropositive persons should be identified as such,” the opposite occurred, i.e., 30% of students agreed or totally agreed. Serrano-Gallardo and Giménez-Maroto already identified such contradictions due to a certain degree of ambiguity in the wording of these items [9]. Although some of the items on the EASE scale could be considered outdated at present due to clinical and cultural changes, we believe that the scale allows general and non-specific attitudes toward HIV/AIDS among nursing students to be explored [25].

Having no religious beliefs was the only analyzed variable that showed an association with better attitudes toward HIV/AIDS, regardless of the influence of the other variables. This result coincides with the findings of other studies, where nursing professionals who practiced a religion, or students who believed religion had a significant role in their lives or firmly thought that the disease etiology was due to divine retribution, showed more stigmatizing attitudes toward their HIV/AIDS patients [20,32,33,34,35].

### Limitations

This study has some limitations. Although the number of students who agreed to answer the questionnaire was higher than the figures published in other studies [1,9,21,22,24,25,28], we believe it could be improved, since students who are more aware of the subject being analyzed could be overrepresented. It is also possible that given the sensitive nature of the subject addressed, when asking for personal opinions, there could have been a social desirability bias that was resolved by guaranteeing the anonymous nature of all participants. Finally, and as we have commented previously, the clinical and cultural changes that have occurred in the last decade with respect to HIV/AIDS could mean that some items on the scale are out of date, although this possible limitation is minimized by establishing a general analysis of attitudes toward HIV/AIDS.

Despite the limitations referred to above, this study contributes current information on the persistence of false opinions regarding HIV/AIDS in a varied sample of nursing students, since four health sciences institutions were analyzed, which were located in different geographic areas but shared the same curriculum. Results may be useful to rethink the type of training our students receive and about the need, therefore, to establish educational content that fosters closer humanization of care and the reduction of HIV/AIDS-associated stigma.

## 5. Conclusions

Nursing students’ attitudes toward HIV/AIDS are relatively positive and get better following the first academic year. When considering all variables of the study, the influence of the academic level waned and the association with religion was maintained. Thus, students with no religious beliefs showed more positive attitudes toward this problem. An improvement in the level of knowledge and experiences among nursing students does not seem to be enough to cause a change of attitude, which may reflect the strong ideological charge that still leads to this health issue today.

It is therefore necessary to carry out a critical analysis of teaching strategies in current nursing programs in order to achieve not only better and more in-depth knowledge of the disease and its modes of transmission, but also a change of attitude, free of negative preconceptions toward HIV/AIDS. This approach would result in a better quality and humanization of the care provided to people with HIV/AIDS. It would also be pertinent in future research to reformulate some of the items on the EASE scale, adapting them to the current reality of people with HIV/AIDS.

## Figures and Tables

**Table 1:** Sociodemographic variables of the sample according to academic year.

Variables	1st Year (*n* = 115)	2nd Year (*n* = 104)	3rd Year (*n* = 86)	4th Year (*n* = 79)	TOTAL (*n* = 384)	*p*
	M (SD)	M (SD)	M (SD)	M (SD)	M (SD)	
**Age (years)**	21.37 (6.34)	22.79 (6.46)	23.71 (6.22)	25.46 (7.57)	23.12 (6.70)	<0.001 **
	***n* (%)**	***n* (%)**	***n* (%)**	***n* (%)**	***n* (%)**	
**Sex**						
Male	15 (13)	19 (18.3)	19 (22.1)	9 (11.4)	62 (16.1)	ns
Female	100 (87)	85 (81.7)	67 (77.9)	70 (88.6)	322 (83.9)	
**Sexual orientation**						
Heterosexual	106 (92.2)	94 (90.4)	76 (88.4)	61 (77.2)	337 (87.8)	0.012 *
Non-heterosexual	9 (7.8)	10 (9.6)	10 (11.6)	18 (22.8)	47 (12.2)	
**Relationship status**						
Not in a relationship	67 (58.3)	60 (57.7)	51 (59.3)	29 (36.7)	207 (53.9)	0.008 **
In a relationship	48 (41.7)	44 (42.3)	35 (40.7)	50 (63.3)	177 (46.1)	
**Religion**						
With religious beliefs	74 (64.3)	71 (68.3)	64 (74.4)	56 (70.9)	265 (69)	ns
With no religious beliefs	41 (35.7)	33 (31.7)	22 (25.6)	23 (29.1)	119 (31)	

M = mean; SD = standard deviation; ns = *p* > 0.05; * = *p* ≤ 0.05; ** = *p* ≤ 0.01.

**Table 2:** Descriptive statistics of the EASE scale according to the sociodemographic variables and academic year.

Variables	M (SD)	Mann–Whitney *U* Test	*p*	Kerby´s
**Sex**		8836.5	ns	
Male	84.52 (8.59)			
Female	85.57 (9.68)			
**Sexual orientation**		9212	ns	
Heterosexual	85.08 (9.45)			
Non-heterosexual	87.7 (9.75)			
**Relationship status**		19,259.5	ns	
Not in a relationship	85.82 (9.58)			
In a relationship	84.91 (9.45)			
**Religion**		13,023.5	0.006 **	0.17
With religious beliefs	84.67 (9.14)			
With no religious beliefs	87.03 (10.14)			
**Kruskal–Wallis *H* Test**				
**Academic year**		8.27	0.041 *	
**Bonferroni Correction**				
First	85.15 (8.33)	7283	0.50 *	−0.22
Second	86.39 (10.86)			
Third	85.58 (8.48)			
Fourth	85.72 (10.23)			
Total	85.25 (9.80)			

M = mean; SD = standard deviation; ns = *p* > 0.05; * = *p* ≤ 0.05; ** = *p* ≤ 0.01.

**Table 3:** Descriptive statistics of the EASE scale items, according to academic year.

	1st Year (*n* = 115)	2nd Year (*n* = 104)	3rd Year (*n* = 86)	4th Year (*n* = 79)	Kruskal–Wallis *H* Test	*p*
	M (SD)	M (SD)	M (SD)	M (SD)		
P1	4.61 (0.99)	4.61 (0.99)	4.62 (1.04)	4.68 (0.96)	0.76	ns
P2	4.11 (0.83)	4.34 (0.95)	4.58 (0.77)	4.46 (0.73)	21.48	<0.001 **
P3	3.87 (1.33)	4.34 (1.09)	4.05 (1.24)	4.03 (1.36)	8.69	0.034 *
P4	4.07 (0.95)	4.31 (0.87)	4.43 (0.83)	4.38 (0.85)	10.85	0.013 *
P5	4.37 (1.06)	4.14 (1.35)	4.06 (1.27)	4.28 (1.09)	3.04	ns
P6	4.57 (0.64)	4.62 (0.77)	4.47 (0.84)	4.52 (0.83)	2.68	ns
P7	4.43 (1.04)	4.19 (1.22)	4.01 (1.27)	4.25 (1.17)	6.9	ns
P8	3.57 (1.27)	3.67 (1.30)	3.6 (1.37)	3.75 (1.25)	1.03	ns
P9	4.29 (0.91)	4.42 (0.9)	4.45 (0.76)	4.25 (1.01)	3.09	ns
P10	2.97 (1.22)	3.19 (1.34)	3.2 (1.26)	3.35 (1.26)	5.21	ns
P11	4.71 (0.69)	4.67 (0.98)	4.7 (0.84)	4.7 (0.82)	1.61	ns
P12	3.8 (1.05)	4 (1.08)	3.91 (1.04)	4.01 (1.15)	3.95	ns
P13	3.62 (1.11)	3.78 (1.12)	4.03 (1.02)	3.78 (1.17)	6.99	ns
P14	2.03 (1.1)	2.12 (1.14)	2.21 (1)	2.15 (1.05)	2.33	ns
P15	4.5 (0.86)	4.43 (1.03)	4.44 (1.08)	4.43 (0.96)	0.73	ns
P16	4 (0.98)	4.36 (0.94)	4.19 (1.00)	4.1 (1.03)	9.57	0.023 *
P17	4.38 (0.94)	4.58 (0.87)	4.47 (0.93)	4.38 (0.97)	4.44	ns
P18	4.57 (0.65)	4.66 (0.71)	4.49 (1.02)	4.44 (0.98)	3.39	ns
P19	4.23 (0.95)	4.38 (0.89)	4.4 (0.77)	4.47 (0.88)	4.39	ns
P20	4 (1.30)	3.94 (1.34)	3.86 (1.31)	3.71 (1.40)	2.75	ns
P21	3.45 (1.28)	3.64 (1.32)	3.43 (1.39)	3.59 (1.32)	1.96	ns

M = mean; SD = standard deviation; ns = *p* > 0.05; * = *p* ≤ 0.05; ** = *p* ≤ 0.01. Definitions of P1–21 can be found in Appendix A.

**Table 4:** Multiple linear regression model for the EASE scale.

Variables	ß	SD	*p*	95% CI	
**Age**	–0.029	0.08	0.704	−0.18	0.12
**Sex**					
Male	Reference				
Female	1.63	1.36	0.228	−1.03	4.30
**Relationship status**					
In a relationship	Reference				
Not in a relationship	0.54	1.03	0.595	−1.48	2.58
**Sexual orientation**					
Heterosexual	Reference				
Non-heterosexual	2.12	1.56	0.171	−0.93	5.18
**Religion**					
With religious beliefs	Reference				
With no religious beliefs	2.15	1.07	0.045b *	0.05	4.26
**Academic year**					
First	Reference				
Second	2.42	1.29	0.060	−0.11	4.95
Third	1.78	1.37	0.194	−0.91	4.47
Fourth	1.60	1.45	0.269	−4.46	1.25

ß = Beta coefficient; * = *p* ≤ 0.05; CI: confidence interval.

## Appendix A

Scale of attitudes toward AIDS for nursing.

1. AIDS does not affect heterosexual couples.
2. Fetuses infected with AIDS should be aborted.
3. There is no risk arising from AIDS carriers’ use of restaurants and pubs.
4. Seropositive women should not be allowed to get pregnant.
5. AIDS is a problem that affects us all.
6. Continued care of an AIDS patient is synonymous with contagion.
7. AIDS carriers are entitled to doctor–patient confidentiality.
8. In daily activities, there is no risk of transmission of HIV.
9. Persons infected with AIDS should be separated from other sick persons.
10. Seropositive persons should be identified as such.
11. Being an AIDS carrier should not be a barrier to accessing education and employment.
12. Specific hospitals for AIDS patients and carriers should be created.
13. AIDS is the greatest affliction of our time.
14. Persons infected with AIDS should be considered victims of the social system.
15. Being an AIDS carrier should not be a barrier to adopting a child.
16. In hospitals, AIDS carriers should not share a room with non-infected persons.
17. I would not like to work with an AIDS carrier.
18. Children who are AIDS carriers should attend special classes.
19. As a precautionary measure, we should avoid contact with AIDS patients and carriers.
20. We should always use gloves when touching someone infected with AIDS.
21. HIV testing should be voluntary and anonymous.
